# A Review on Maternal Parenting, Child's Growth Stunting, and Oral Health

**DOI:** 10.1055/s-0043-1764428

**Published:** 2023-04-27

**Authors:** Rasyid Abdulaziz, Netty Suryanti, Arlette Suzy Setiawan

**Affiliations:** 1 Faculty of Dentistry, Universitas Padjadjaran, Bandung, Indonesia; 2 Department of Community Dentistry, Faculty of Dentistry, Universitas Padjadjaran, Bandung, Indonesia; 3 Department of Pediatric Dentistry, Faculty of Dentistry, Universitas Padjadjaran, Bandung, Indonesia

**Keywords:** stunting, mother, feeding practice, oral health, early childhood caries

## Abstract

Stunting has gained global attention as one of the most critical problems in public health. As the first and dominant figure in a child's life, the mother is responsible for determining the proper parenting behaviors to apply to maintain the child's physical health. Stunting is often associated with early childhood caries (ECC) and molar incisor hypomineralization, which can be manifested into each other through various mechanisms. Therefore, it is crucial to explore how far maternal parenting behaviors affect stunting and oral health. This study aims to determine which maternal parenting behaviors can affect stunting and oral health. A systematic search was used through PubMed and Google Scholar to search for published articles between 2011 and 2021. The articles analyze maternal parenting behaviors with stunting and poor oral health. Final analysis was used on 21 articles containing 18 cross-sectional studies, 2 cohort studies, and 1 randomized controlled trial. The result implied that the high prevalence of stunting and ECC is the combined result of prolonged breastfeeding practices (7 articles), poor complementary feeding practice (6 articles), high consumption of sugar (5 articles), and poor oral hygiene practices (5 articles). Maternal parenting styles in the aspect of fulfilling nutrition and maintaining oral health affect the occurrence of stunting and ECC in children.

## Introduction

Growth stunting is a form of linear growth retardation manifested in children's inability to achieve their optimal height compared to the average standard height. 1 The World Health Organization (WHO) defines stunting as an impairment of a child's growth and development due to malnutrition, recurrent infections, and insufficient psychosocial stimulation. Children were defined as stunted if their age-related height was more than two standard deviations below the WHO Child Growth Standards median. 2 It is estimated that one in four children under the age of 5 worldwide experiences stunted growth. Stunting occurs from the prenatal period until 2 years old. Stunting results from complex interactions between family, environment, and socioeconomic and sociocultural conditions. 3 Family has a vital role in the parenting styles. 4 5

The parenting style is defined as a family's ability to give time, attention, and support to fulfil children's physical, psychological, and social needs. 6 Maternal parenting styles are affected by internal and external factors. Maternal knowledge and attitude are the most crucial for the proper practices for their children. 7 8 Maternal knowledge can affect their daily habits in preserving children's health, especially their nutritional status. Stunting happens when there are chronic nutritional deficiencies in early childhood life. Mothers are responsible for facilitating their children's nutrition through their parenting styles. Maternal parenting styles also play a significant role in children's oral health. 9 Early childhood life provides chances for a mother to be able to build their children's oral hygiene behavior. 10

Poor oral health can affect nutritional status through various problems linked with certain parenting styles. Oral hygiene practice taught since early childhood has been proven to effectively prevent inflammation diseases in the oral cavity, such as early childhood caries (ECC). Nutritional deficiency could happen in children with ECC because of inadequate nutrition intake, which can lead to malnutrition. 11 12 There is a correlation between caries and protein consumption rate in children. ECC may cause mouth pain and loss of appetite, and affect mastication and nutrient absorption. The chronic nutritional deficiency can lead to some nutritional status problems and increase the risk of stunting. 13 In addition, stunting can also be associated with the presence of molar incisor hypomineralization (MIH), which occurs during the process of tooth formation in the uterus. 14

WHO recommends appropriate infant and young child feeding (IYCF) practices to fulfil nutritional needs in early childhood. 15 Adequate nutrition has a vital role in oral health development and protection. Micronutrients, protein, and vitamin deficiencies in children with malnutrition can cause abnormality in the oral cavity. 16 The development of teeth in the pre-eruptive phase is impacted by the nutritional status. Caries-related malnutrition can occur in early childhood through mechanisms such as enamel hypoplasia and hyposalivation. 17 18 Black et al have linked malnutrition with periodontal diseases such as necrotizing gingivitis through the deficiency of vitamin B complex and zinc, followed by inadequate oral hygiene. 19

Nutritional deficiency can cause tongue and oral mucosae diseases, such as aphthous stomatitis and atrophic glossitis. 16 Based on the description above, stunting and oral health have a strong potential correlation with maternal parenting styles. Therefore, this study aimed to determine which maternal parenting styles cause a child's stunting and poor oral health.

## Methods

A systematic literature search was conducted to identify which maternal parenting styles affect stunting and oral health in a systematic literature review research design according to the Preferred Reporting Items for Systematic Reviews and Meta-Analyses (PRISMA) ^20^ guidelines, and it is a frequent reference in data identification, extraction, and evaluation. The results data was documented using Mendeley.

### Search Strategy

A literature search was conducted using the electronic database (PubMed). Keywords used for this review were related to the nutritional status of stunting (Stunting/Chronic Malnutrition/Malnutrition/Nutritional Status), maternal parenting styles (Mother/Maternal Parenting Styles/Maternal Knowledge/Maternal Attitude/Maternal Practice/Feeding Practice/Oral Hygiene Practice), and oral health of children (Oral Health/Caries/Early Childhood Caries/Periodontal/Gingiva/Dental). The articles were identified by applying the inclusion and exclusion criteria to determine the selected sample.

### Inclusion Criteria and Exclusion Criteria

The articles selected in this review were based on the following inclusion criteria: studies conducted in humans, subjected to infants and preschool children (0–5 years), published in the last 10 years between March 2011 and March 2021, and published in Bahasa Indonesia and English, available in full-text, including the population with stunting and the maternal parenting styles in fulfilling the nutritional aspect of children and in maintaining oral health of children. All of the unmatched articles were excluded from the study.

### Quality Appraisal

The researcher assessed and appraised the quality of the articles using the National Institutes of Health (NIH) Quality Assessment Tool. The quality of the articles was assessed with a scoring system determined by specific criteria. The criteria were presented in several questions. Articles that scored 10 to 14 were classified as “good,” those with a score of 5 to 9 were considered “fair,” and those with a score of 0 to 4 were considered “poor.” Quality assessment of the articles was done by the first author and with a random sampling technique by two reviewers. Finally, the results were discussed to reach a consensus.

### Data Extraction

The extracted studies were summarized in several tables: general characteristics of 21 articles, prevalence of stunting and oral health problems in the sample, the analysis results of maternal parenting behaviors, findings in maternal breastfeeding and feeding practices, findings in maternal oral hygiene practices, and quality appraisal.

## Results

### Study Selection Results

**Fig. 1:**
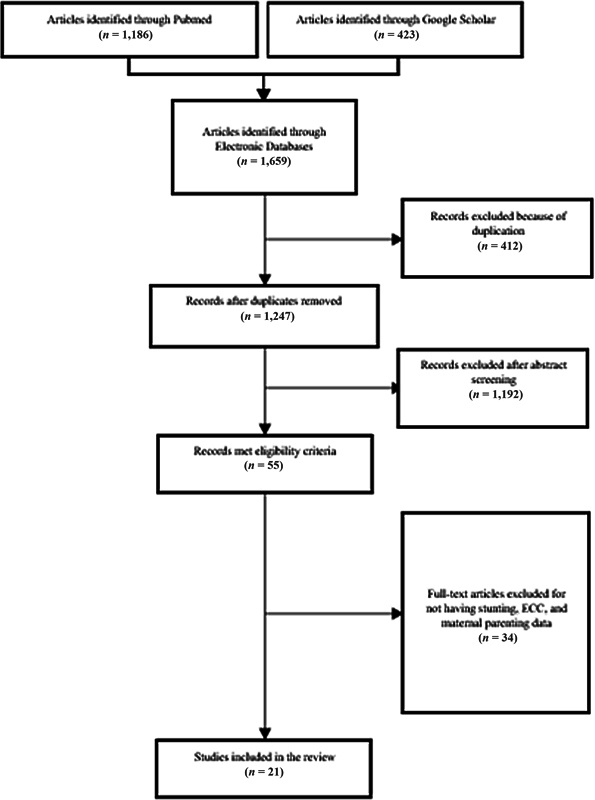
Flow diagram of the selection process.

Fig. 1 illustrates the flow diagram for the study selection. A literature search conducted in July 2021 through electronic databases, which yielded 1,659 articles. Of these, 412 articles were duplicates and excluded during the title screening process. While screening the titles and abstracts, 1,192 more articles were excluded for failing to meet the inclusion criteria. A total of 55 articles met our eligibility criteria; however, only 21 were included in the review. The other 34 articles were not included due to lack of the nutritional status, oral health status, and maternal parenting practices.

### Characteristics of the Included Study

The general characteristics of the 21 articles are described in Table 1 . Most of the studies included in this review were conducted in the Asian continent. One article each ( *n* = 1) was from Cambodia, India, and Bangladesh, and two articles each ( *n* = 2) were from Nepal, China, and Vietnam. 7 21 22 23 24 25 26 27 28 Seven ( *n* = 7) studies were from Africa: one each ( *n* = 1) from Tanzania, Nigeria, Kenya, Malawi, and Uganda, and two articles ( *n* = 2) were from Ethiopia. 21 29 30 31 32 33 34 Five ( *n* = 5) studies were conducted in the American continent: one each ( = 1) in El Salvador and Mexico, and three ( *n* = 3) in Ecuador. 35 36 37 38 39 Most of the selected studies ( *n* = 18) followed a cross-sectional study design, while two studies ( *n* = 2) used a cohort study design, and one study ( *n* = 1) used a randomized controlled trial. The data were measured with questionnaires, interviews, and clinical examinations, including intraoral and anthropometric examinations.

**Table 1:** General characteristics of 21 articles

No.	Title	Author (country, year)	Research design and sample size	Objective	Measurement method	Conclusion
1.	Stunting malnutrition associated with severe tooth decay in Cambodian toddlers	Renggli et al (Cambodia, 2021)	Cohort 1,307 pairs mother-child	To examine the relationship between severe dental caries and anthropometric changes over a 1-year period	• Questionnaire • Anthropometric measurement • Intraoral examination	The study highlights the need to prevent and treat early childhood tooth decay as an important part of programs to prevent child undernutrition as well as noncommunicable diseases (NCDs), and to promote children's optimal growth and development during a critical stage of life
2.	Prevalence of early childhood caries, risk factors and nutritional status among 3-5-year-old preschool children in Kisarawe, Tanzania	Ndekero et al (Tanzania, 2021)	Cross-sectional 831 mother–child pairs	To assess the association between anthropometric measures indicated by weight-for-age and early childhood caries (ECC) using longitudinal analyses	• Questionnaire • Anthropometric measurement • Intraoral examination	This study demonstrated a significantly negative relationship between ECC and children's anthropometric measures indicated by weight-for-age, and positive relationship with sugar exposure and poor oral hygiene indicated by visible plaque on upper anterior teeth
3.	Early childhood junk food consumption, severe dental caries, and undernutrition: a mixed-methods study from Mumbai, India	Athavale et al (India, 2020)	Cross-sectional 959 mother–child pairs	To use a mixed-method approach to describe the risk factors for ECC and associations with malnutrition in a convenience sample of children and families from low-income urban communities in Mumbai, India	• Survey • Anthropometric measurement • Intraoral examination • Focus group discussion (FGD)	This study found daily consumption of junk food, progressing frequency and severity of untreated ECC from infancy to age 6, frequent mouth pain, and association between severe ECC and undernutrition
4.	Associations between child snack and beverage consumption, severe dental caries, and malnutrition in Nepal	Zahid et al (Nepal, 2020)	Cross-sectional 273 mother–child pairs	To explore the associations between Nepali children's dietary consumption habits and dental caries and between severe caries and nutritional status	• Interview • Anthropometric measurement • Intraoral examination	This cross-sectional study demonstrated a high prevalence of the daily consumption of junk food and sugar-sweetened beverages, signiﬁcant associations between the frequency of junk food consumption and severe dental caries, and signiﬁcant associations between severe dental caries and the risk for malnutrition
5.	Association between nutritional status and early childhood caries risk profile in a suburban Nigeria community	Folayan et al (Nigeria, 2020)	Cross-sectional 1,439 mother–child pairs	To determine the association between malnutrition and ECC in a suburban population in Nigeria	• Questionnaire • Anthropometric measurement • Intraoral examination	The study has furthered our understanding of the association between ECC and malnutrition with a different disease mix: high prevalence of malnutrition and low prevalence of ECC
6.	Dietary intake and prevalence of dental caries among five-year-old children in urban and rural areas of Uasin-Gishu County, Kenya	Wakhungu et al (Kenya, 2020)	Cross-sectional 382 mother–child pairs	To assess the dietary intake and prevalence of dental caries among 5-year-old children in urban and rural areas of Uasin-Gishu County, Kenya	• Questionnaire • Anthropometric measurement • Intraoral examination	There is inadequate intake of energy, vitamin A, and Iron in the diet consumed by the 5-year-old in both the urban and rural areas of Uasin-Gishu County, Kenya
7.	Sugary liquids in the baby bottle: risk for child undernutrition and severe tooth decay in rural El Salvador	Achalu et al (El Salvador, 2020)	Cross-sectional 797 mother–child pairs	To identify dietary, oral health, and sociodemographic risk factors for child undernutrition and severe early childhood caries (sECC) for rural communities in El Salvador	• Questionnaire • Anthropometric measurement • Intraoral examination	High rates of feeding young children sugar-sweetened beverages in the baby bottle were associated with increased risk of developing undernutrition and ECC, and ECC was further associated as a risk factor for undernutrition
8.	Undernutrition is associated with change in severe dental caries	Shen et al (China, 2020)	Cross-sectional 1,111 mother–child pairs	To examine the association of baseline prevalence of thinness or stunting and the incidence of dental caries among preschool children in Liaoning Province, China	• Questionnaire • Anthropometric measurement • Intraoral examination	While undernutrition indicated by stunting appeared to be a risk factor for severe dental caries, it could also be a marker for other determinants of progress of dental caries including socioeconomic factors and lack of use of dental services
9.	The bidirectional relationship between weight, height, and dental caries among preschool children in China	Shen et al (China, 2020)	Cross-sectional 1,320 mother–child pairs	To assess whether there is an association between dental caries at baseline and change in weight and height, and whether there is an association between weight and height at baseline and changes in dental caries	• Questionnaire • Anthropometric measurement • Intraoral examination	The study demonstrated a significantly negative relationship between baseline dental caries and children growth indicated by height-for-age (HAZ), and between baseline weight and caries increment
10.	Early childhood oral health and nutrition in urban and rural Nepal	Tsang et al (Nepal, 2019)	Cross-sectional 836 mother–child pairs	To examine the socio-behavioral risk factors for ECC in rural and urban populations and associations between caries and malnutrition in Nepali children to help guide interventions to improve children's health and well-being	• Questionnaire • Anthropometric measurement • Intraoral examination	This study provides evidence that families in urban and rural Nepal are experiencing a nutrition transition in which junk food has become a daily staple of young children's diet, contributing to high rates of ECC, mouth pain, and malnutrition
11.	Maternal determinants of optimal breastfeeding and complementary feeding and their association with child undernutrition in Malawi	Walters et al (Malawi, 2019)	Cross-sectional 3,168 mother–child pairs	(1) To determine the situation of breastfeeding and complementary feeding practices in Malawi, (2) to identify the maternal determinants of each infant and young child feeding (IYCF) indicator, and (3) to analyze the association between each IYCF indicator and stunting, underweight, and wasting	• Questionnaire • Anthropometric measurement	The findings from this study show that Malawian children (aged 13–23 mo) meeting minimum meal frequency (MMF) or minimum acceptable diet (MAD) recommendations were less likely to be underweight
12.	Maternal nutrition counselling is associated with reduced stunting prevalence and improved feeding practices in early childhood a post program comparison study	Mistry et al (Bangladesh, 2019)	Cross-sectional 1,452 mother–child pairs	To assess the impact of this intervention on the prevalence of stunting and feeding practices among children younger than 5 y	• Questionnaire • Anthropometric measurement	The present study suggested that counselling mothers on child feeding practices may be effective in reducing the prevalence of stunting among children younger than 5 y
13.	Maternal and child nutrition and oral health in urban Vietnam	Huang et al (Vietnam, 2019)	Cross-sectional 571 mother–child pairs	To present baseline nutrition and oral health data on a convenience sample of children aged 2–5 y and their mothers/caregivers participating in a preventive school-based study designed to understand child nutrition and oral health	• Questionnaire • Anthropometric measurement • Intraoral examination	This sample of children aged 2–5 y from two urban/peri-urban regions of Vietnam found evidence of the global nutrition transition and its adverse impact on child nutrition and oral health
14.	Associations of childhood, maternal and household dietary patterns with childhood stunting in Ethiopia: proposing an alternative and plausible dietary analysis method to dietary diversity scores	Melaku et al (Ethiopia, 2018)	Cross-sectional 2,950 mother–child pairs	To identify household, maternal and child dietary patterns and investigate their associations with childhood stunting in Ethiopia using the same dietary data collected for determining dietary diversity score (DDS)	• Questionnaire • Anthropometric measurement	DDSs are not significantly associated with HAZ (stunting), a dietary pattern characterized by a high intake of dairy, vegetables, and fruits by households, mothers, and children is positively associated with HAZ and inversely associated with stunting
15.	Effects of nutrition and hygiene education on oral health and growth among toddlers in Rural Uganda: Follow-up of a cluster-randomised controlled trial	Muhoozi et al (Uganda, 2018)	Randomized controlled trial 248 mother–child pairs	To examine whether (1) the education intervention had an impact on oral health behavior and early onset of caries and (2) whether early onset of caries was related to the nutritional status, a proxy for growth, of the children	• Questionnaire • Anthropometric measurement • Intraoral examination	We found that an education intervention improved oral hygiene, which prevented the development and progression of caries
16.	Early childhood dental caries, mouth pain, and malnutrition in Ecuadorian Amazon Region	So et al (Ecuador, 2017)	Cross-sectional 1,407 mother–child pairs	To examine the relationships between ECC, parent-reported mouth pain, and nutritional status among participating children in the Kichwa community	• Questionnaire • Anthropometric measurement • Intraoral examination	In a population with a high prevalence of severe dental caries and malnutrition, parent-reported mouth pain can reliably predict the presence of severe caries in young children, and parent-reported mouth pain interfering with sleeping can reliably predict poor nutritional status in children. This relationship seems to be most salient for children aged 3–6 y
17.	Infant and young child feeding practices and stunting in two highland provinces in Ecuador	Roche et al (Ecuador, 2017)	Cross-sectional 293 mother–child pairs	To assess the feeding practices and nutritional status of infants and young children in high-altitude communities of the Ecuadorian Andes	• Questionnaire • Anthropometric measurement	Mothers should be encouraged in their positive practice of continued breastfeeding. Timely introduction of complementary foods and access to iron-rich foods may need urgent attention. Healthy growth will require improved access and use of health services and improved hygiene to reduce the burden of illness
18.	Early childhood caries and malnutrition: baseline and two-year follow-up result of a community-based prevention intervention in Rural Ecuador	Sokal-Gutierrez et al (Ecuador, 2016)	Cohort 731 mother–child pairs	To examine the cross-sectional and longitudinal relationship between ECC and malnutrition in the context of a community-based intervention designed to prevent ECC and malnutrition, starting from birth	• Questionnaire • Anthropometric measurement • Intraoral examination	Our cross-sectional and longitudinal analyses demonstrated that the children who began the intervention in the first 2 y of life experienced the greatest benefits. This study underscores the importance of universal, community-wide programs to jointly address oral health and nutrition
19.	Poor breastfeeding, complementary feeding and dietary diversity in children and their relationship with stunting in rural communities	Cortes et al (Meksiko, 2016)	Cross-sectional 189 mother–child pairs	To characterize feeding practices in children under 2 y of age in rural communities of Hidalgo and determine their relationship with stunting	• Questionnaire • Anthropometric measurement	Maternal education on breastfeeding and complementary feeding (CF) as well as improvements in childhood dietary diversity has been found to be a major factor in reducing stunting
20.	Early childhood caries, mouth pain, and nutritional threats in Vietnam	Khanh et al (Vietnam, 2015)	Cross-sectional 593 mother–child pairs	To investigate the relationships among ECC, mouth pain, and nutritional status in children aged 1–6 y in Southern and Central Vietnam	• Questionnaire • Anthropometric measurement • Intraoral examination	ECC might negatively affect children's nutritional status, which might be mediated by the depth of decay, chronic inﬂammation, and mouth pain. Family-based and prevention-oriented nutrition and oral health programs are needed and should start during pregnancy and infancy
21.	Feeding patterns and stunting during early childhood in rural communities of Sidama, South Ethiopia	Tessema et al (Ethiopia, 2013)	Cross-sectional 575 mother–child pairs	To assess feeding practices of children younger than 2 y of age, household food security status, and their association with stunting in selected rural communities of Sidama, Southern Ethiopia	• Questionnaire • Anthropometric measurement	The feeding practices of most mothers did not meet WHO recommendations. Behavior change communication about the importance of optimal complementary feeding and antenatal care (ANC) attendance should be strengthened through extensive use of the Health Extension Workers to reduce the level of child stunting in the study area

### Prevalence of Stunting and ECC

The prevalence of stunting and ECC are shown in Table 2 . All of the studies stated a prevalence of stunting with various rates. The highest prevalence of stunting was from a study in India (41.8%). 40 Studies found the prevalence of ECC with the highest rates in Vietnam (46.8%). 27 Other oral health problems indicators were mouth pain ( *n* = 8), poor Oral Hygiene Index (OHI; *n* = 1), poor PUFA Index ( *n* = 1), and poor Visible Plaque Surface Index (VPSI; *n* = 1). 21 26 27 28 29 33 35 36 38

**Table 2:** Prevalence of stunting and oral health problems in the sample

Author citations	Sample size ( *n* )	Stunting		ECC		Other oral health indicators		
		%	*n*	%	*n*	*x*	%	*n*
Renggli et al (2021)	1,307	25.6 39.9	521	51.9 63.6	831	–	–	–
Ndekero et al (2021)	831	1.6	12	44.8	372	VPSI	70.4	262
Athavale et al (2020)	959	41.8	401	49.6	476	MP	27.2	243
Zahid et al (2020)	273	21	57	58	158	PUFA	20	54
Folayan et al (2020)	1,539	54.7	848	4.3	66	OHI-S	3.9	60
Wakhungu et al (2020)	382	14.6	55	39.2	150	–	–	–
Achalu et al (2020)	797	10.4	82	47.4	377	MP	19.3	153
Shen et al (2020)	1,111 772	4.92	38	5.18	40	–	–	–
Shen et al (2020)	1,320 772	4.9 6.9	38	3.184.21	40	–	–	–
Tsang et al (2019)	836	34.2	285	58.2	486	MP	20	167
Walters et al (2019)	3,168	30.8	975	–	–	–	–	–
Mistry et al (2019)	1,452	37.19	539	–	–	–	–	–
Huang et al (2019)	571	4.2	24	74.6	425	MP	56.3	321
Melaku et al (2018)	2,950 3,788	38.5	1,458	–	–	–	–	–
Muhoozi et al (2018)	248 196	28 28.6	70 56	27.8	47	MP	25.1	47
So et al (2017)	1,407	35.9	505	65.4	920	MP	33.8	475
Roche et al (2017)	293	56	165	–	–	–	–	–
Sokal-Gutierrez et al (2016)	731 658 698	43 34.7	314 242	39.2 36.6	287 256	MP	30.2 20.8	221 145
Cortes et al (2016)	189	31.9	60	–	–	–	–	–
Khanh et. al (2015)	593	4.2	24	74.4	441	MP	47.1	268
Tessema et al (2013)	575	37.2	214	–	–	–	–	–

Abbreviations: MP, mouth pain; OHI-S, Oral Hygiene Index Simplified; PUFA, the PUFA Index; VPSI, Visible Plaque Surface Index.

### Maternal Parenting Styles

The prevalence of each maternal parenting style variables, including breastfeeding, feeding, and oral hygiene practices, are covered in Table 3 . The summary of maternal practices in IYCF is described in Table 4 , and the summary of maternal oral hygiene practices is described in Table 5 .

**Table 3:** Analysis result of maternal parenting behaviors

Indicators		Prevalence on each article (%)																				
		1	2	3	4	5	6	7	8	9	10	11	12	13	14	15	16	17	18	19	20	21
Breastfeeding practices	Initiation of breastfeeding within 1 h of birth											78	84.8				51.8					31.5
	Exclusive breastfeeding up to 6 mo								49	49		60	89.99		72.4					48.2		
	Prolonged breastfeeding up to 1 y			92.9				94.3			98.5	89		95.3		69.4			97.6	39		
Feeding practices	Good knowledge										85.5									85.2		25.6
	Initiation of complementary feeding by 6 mo	15.4											90.3			95.2				30		20.3
	Minimum dietary diversity (MDD)											32	80		6.3		49.1					6
	Minimum meal frequency (MMF)											23										24.5
	Minimum acceptable diet (MAD)	49										12						71.4				4.3
	Iron-rich foods consumption											12						57.8				
	High-sugary intakes		15.4	52.4	60	14	33.5	73		97	50.8			11.9			27.4		42.4		11.8	
Oral health practice	Good knowledge			75.2				12.9			26.1			67.5								
	Positive oral health perception			79.9				38.9			80.3			91.1		90.7						
	Mother help with brushing		35.5	63.2							21.9			80.7		52.7	51.8		78.7			
	Frequent tooth brushing twice a day		23.1						36.8	36.8						46.6						
	Toothpaste containing fluoride		81.7	91.7							90.4											
	Toothbrush sanitation			80.7							74.4			99.1		35.4						
	Dentist appointment			14				16.6	14.25	14.25	10.3			44.4		4.1	49.1		66.1			

Note: 1, Renggli et al (2021); 2, Ndekero et al (2021); 3, Athavale et al (2020); 4, Zahid et al (2020); 5, Folayan et al (2020); 6, Wakhungu et al (2020); 7, Achalu et al (2020); 8, Shen et al (2020); 9, Shen et al (2020); 10, Tsang et al (2019); 11, Walters et al (2019); 12, Mistry et al (2019); 13, Huang et al (2019); 14, Melaku et al (2018); 15, Muhoozi et al (2018); 16, So et al (2017); 17, Roche et al (2017); 18, Sokal-Gutierrez et al (2016); 19, Cortes et al (2016); 20, Khanh et al (2015); 21, Tessema et al (2013).

**Table 4:** Findings in maternal breastfeeding and feeding practices

Nutrition practices		Positive	Negative	Conclusion
Breastfeeding practices	Initiation of breastfeeding within 1 h of birth	Walters et al; Mistry et al; So et al	Tessema et al	Majority of the mothers were doing an early initiation of breastfeeding
	Exclusive breastfeeding up to 6 mo	Shen et al; Walters et al; Mistry et al; Melaku et al; Cortes et al	–	Majority of the mothers were doing an exclusive breastfeeding practice
	Prolonged breastfeeding up to 1 y	Athavale et al; Achalu et al; Tsang et al; Walters et al; Huang et al; Muhoozi et al; Sokal-Gutierrez et al	Cortes et al	Majority of the mothers were doing a prolonged breastfeeding practice
Feeding practices	Initiation of complementary feeding by 6 mo	Mistry et al; Muhoozi et al	Renggli et al; Cortes et al; Tessema et al	Majority of the mothers were doing a poor complementary feeding practice
	High sugary intakes	Athavale et al; Zahid et al; Achalu et al; Shen et al; Tsang et al	–	Majority of the mothers were giving a high sugar intakes to their children

**Table 5:** Findings in maternal oral hygiene practices

Oral health practices	Positive	Negative	Conclusion
Good oral hygiene practices knowledge	Athavale et al; Huang et al	Achalu et.al; Tsang et al	Half of the mothers were having a poor oral health knowledge
Positive oral health perception	Athavale et al; Tsang et al; Huang et al; Muhoozi et al	Achalu et al	Majority of the mothers were having a positive perception regarding oral health
Mother help with brushing	Athavale et al; Shen et al; Huang et al; Muhoozi et al; So et al	Ndekero et al; Tsang et al	Majority of the mothers were helping their children in toothbrushing
Frequent tooth brushing twice a day	–	Ndekero et al; Shen et al; Muhoozi et al	Majority of the mothers were doing inadequate frequency of daily toothbrushing
Toothpaste containing fluoride	Ndekero et al; Athavale et al; Tsang et al	–	Majority of the mothers were using a flour-based toothpaste
Toothbrush sanitation	Athavale et al; Tsang et al; Huang et al	Muhoozi et al	Majority of the mothers were capable to maintain the sanitation
Dentist appointment	Sokal-Gutierrez et al	Athavale et al; Achalu et al; Shen et al; Tsang et al; Muhoozi et al; So et al	Majority of the mothers were not doing a dental appointment for their children

### Maternal Breastfeeding Practices

Fourteen studies ( *n* = 14) identified maternal breastfeeding practices for their children. Most of the studies ( *n* = 12) stated good breastfeeding practices, including prolonged breastfeeding ( *n* = 7), early initiation, and exclusive breastfeeding ( *n* = 4). However, Tessema et al 34 and Cortes et al 39 found poor breastfeeding practices among their population.

Table 4 shows that most of the mothers in most studies initiated early breastfeeding and practiced exclusive and prolonged breastfeeding.

### Maternal Feeding Practices

All of the selected studies examined the maternal feeding practices among the study population. Six studies ( *n* = 6) found poor initiation and diversity of complementary foods. 18 27 30 32 35 37 Five studies ( *n* = 5) found high sugar consumption, with the highest prevalence rates from Athavale et al (52.4%). 7 20 21 24 34 Table 4 shows that mothers had shown poor complementary feeding practices and high intake of sugary foods for their children.

### Maternal Oral Hygiene Practices

The majority of the studies ( *n* = 16) analyzed the maternal oral hygiene practices. 7 18 19 20 21 22 23 24 25 26 29 31 32 33 34 36 Four studies ( *n* = 4) examined the positive perception of mothers in their children's oral health. 7 20 29 31 Two studies ( *n* = 2) showed a low maternal knowledge in maintaining oral health. 7 24 Three studies ( *n* = 3) showed inadequate toothbrushing frequency below twice daily. 19 25 31 Five studies ( *n* = 5) showed that mothers helped their children to brush their teeth. 20 25 29 31 32 Three studies ( *n* = 3) stated that mothers used fluor-based toothpaste. 7 19 20 Three studies ( *n* = 3) found good sanitation. 7 20 29 Six studies ( *n* = 6) examined the low rates of dental appointments. 7 20 24 25 31 32

Table 5 shows that most of the mothers in the studies had a good perception of their children's oral health, helping them brush their teeth, used flour-based toothpaste, and maintained good sanitation. However, other findings stated that half of the mothers do not have good knowledge about oral health, which does not imply proper oral hygiene practice with low frequency of daily toothbrushing and low dental appointment.

### Quality Appraisal

The quality assessment of the articles included in this review is summarized in Table 6 . Of the 21 studies ( *n* = 21) reviewed, 18 were classified as “good” and 3 as “fair” with a score of 9 to 14. This quality assessment showed a good result, which indicates the high quality of the article.

**Table 6:** Quality appraisal

Study	NIH quality assessment tools criteria														Quality (score)
	Q1	Q2	Q3	Q4	Q5	Q6	Q7	Q8	Q9	Q10	Q11	Q12	Q13	Q14	
Renggli et al (2021)	v	v	v	v	v	v	v	v	v	v	–	v	v	v	Good (13)
Ndekero et al (2021)	v	v	v	v	v	v	v	v	–	–	v	–	–	–	Fair (9)
Athavale et al (2020)	v	v	v	v	v	–	v	v	v	–	–	v	v	v	Good (11)
Zahid et al (2020)	v	v	v	v	v	–	v	v	v	v	v	v	–	v	Fair (12)
Folayan et al (2020)	v	v	v	v	v	v	v	v	v	v	v	v	v	v	Good (14)
Wakhungu et al (2020)	v	v	v	v	v	v	v	v	v	–	v	v	v	v	Good (13)
Achalu et al (2020)	v	v	v	v	v	–	v	v	v	–	v	v	v	v	Good (12)
Shen et al (2020)	v	v	v	v	v	–	v	v	–	v	–	–	v	–	Fair (9)
Shen et al (2020)	v	v	v	v	v	–	v	v	–	v	–	–	v	–	Fair (9)
Tsang et al (2019)	v	v	v	v	v	v	v	v	v	v	v	–	v	v	Good (13)
Walters et al (2019)	v	v	v	v	v	v	v	v	v	v	–	v	v	v	Good (13)
Mistry et al (2019)	v	v	v	v	v	–	v	v	–	v	–	v	v	–	Good (11)
Huang et al (2019)	v	v	v	v	v	–	v	v	–	v	v	v	v	v	Good (12)
Melaku et al (2018)	v	v	v	v	v	v	v	v	v	v	v	v	v	–	Good (13)
Muhoozi et al (2018)	v	v	v	v	v	v	v	v	v	v	v	v	v	v	Good (14)
So et al (2017)	v	v	v	v	v	v	v	v	v	v	v	v	v	v	Good (14)
Roche et al (2017)	v	v	v	v	v	v	v	v	v	v	–	v	–	v	Good (12)
Sokal-Gutierrez et al (2016)	v	v	v	v	v	v	v	v	v	v	v	v	–	v	Good (13)
Cortes et al (2016)	v	v	v	v	v	–	v	v	–	v	–	v	–	v	Good (10)
Khanh et al (2015)	v	v	v	v	v	v	v	v	v	v	v	–	v	–	Good (12)
Tessema et al (2013)	v	v	v	v	v	v	v	v	v	v	v	–	v	v	Good (13)

Note: Q1. Was the research question clearly stated?

Q2. Was study population clearly and fully described?

Q3. Were the participating rates more than 50%?

Q4. Were the subjects comparable?

Q5. Was the sample being assessed?

Q6. Was there a measurement for independent and dependent variables?

Q7. Was the time range clearly stated?

Q8. Were outcome measures clearly defined, valid, reliable, and implemented consistently across all study participants?

Q9. Was the measurement of independent variable clearly stated?

Q10. Was the exposure implemented more than once?

Q11. Was the measurement of dependent variable clearly stated?

Q12. Was the outcome blinded by the exposure?

Q13. Was the loss from baseline to follow-up more than 20%?

Q14. Was the key potential being measured statistically to define a correlation?

Good: Met 10–14 criteria; Fair: Met 5–9 criteria; Poor: Met 0–4 criteria.

“–“ means not applicable or not reported. “v” indicates check.

## Discussion

Our review summarized previously published studies investigating maternal parenting styles and their association with the prevalence of stunting and ECC. Most studies found a high prevalence of stunting and ECC, with the highest prevalence among developing countries. These findings are linked to the low economic and educational status found in the mothers. Low socioeconomic status is one of the most significant risk factors for stunting and ECC. 17

Breastfeeding practices hold an essential role in early childhood nutrition. It is the first food consumed by children since children, and it becomes a nutritional status indicator at the age of 0 to 2 years. 41 Exclusive breastfeeding in the early 6 months is needed to gain optimal growth, development, and health of the children. 39 Breastfeeding practices strongly correlate with stunting conditions in children below the age of 2 years. Most studies found a prolonged breastfeeding practice for up to 1 year. According to WHO, prolonged breastfeeding is may prevent stunting. 2 42

This finding was contrary to the high prevalence of stunting, which can also be found in this review. 21 22 23 24 25 26 30 31 35 40 43 Another optimal breastfeeding indicator has its role in linking this phenomenon. Prolonged breastfeeding is a complex behavior that cannot be separated from other aspects such as good frequency, method, and time. These data cannot be found in all of the studies, resulting in lack of information regarding the proper breastfeeding practice. 7 24 25 26 27 31 32 33 35 40 Problems during breastfeeding that lead to malnutrition could be screened by methods as the following: ask the mothers if there is a problem during breastfeeding, scanty breast milk, or appetite loss in children younger than 2 years. In the childhood group, simple and valid nutritional screening tools were the following: loss of appetite, skipping of meals and watching TV and videotapes and playing computer games for more than 2 hours per day. 44

The initiation of complementary feeding at 6 months was a supporting effort to fulfil early childhood nutritional needs. The combination of adequate breastfeeding and feeding practices was needed to meet the improved nutritional needs of children, so the breastfeeding practice was no longer the only nutritional source. Studies in this review have shown inadequate complementary feeding practices. Most mothers had practiced prolonged breastfeeding without properly introducing complementary feeding practices. This can be another potential explanation for determining the relationship between prolonged breastfeeding practice and the occurrence of stunting. 45 This explanation was supported by Cetthakrikul et al, 46 who found prolonged breastfeeding in stunted children in Thailand. This behavior was linked to extreme poverty, which was an illustration of the low socioeconomic status of the population. The economic obstacles led the mothers to practice prolonged breastfeeding without pursuing proper complementary feeding practices to reduce consumption costs. 7 24 25 29 Prolonged breastfeeding can cause nutritional deficiency only if proper feeding is not initiated after 6 months. 46

Prolonged breastfeeding was also linked to high occurrence of ECC in this review. This relationship was supported by a systematic review by Tham et al 47 who found that breastfeeding for up to 12 months increased the risk of caries. This could be because the teeth have started to erupt at this age, and the risk of caries also begins to occur. 48 Other factors linked with prolonged breastfeeding practice were habits of nocturnal feeding, consumption of cariogenic foods, and poor oral hygiene. Prolonged breastfeeding also offers some protection from certain diseases. Hermont et al found in their systematic review that breastfeeding practice for up to 12 months can prevent malocclusions such as disto-occlusion, crossbite, and open bite. 49 Socioeconomic status has many associations beyond malnutrition. For example, the study by Ibrahim et al showed higher percentages of caries in families of lower socioeconomic status. MIH, as is ECC due to malnutrition in children, is higher in populations with lower socioeconomic status in some countries. 14

Optimal feeding practice refers to an acceptable IYCF practice recommended by WHO. This indicator illustrates the importance of early childhood nutrition to facilitate rapid growth and development in early childhood. This review found a relationship between mothers' inadequate complementary feeding practices and occurrence of caries in stunted children. 48 Frequent feeding practices become a determinant factor in energy sufficiency. At the same time, diet variation has a role in determining the quality of nutrition. 15 The diet diversity in this review was considered low and impacted the nutritional and oral health status. An excellent complementary feeding practice relies on diverse nutrition intakes such as meats, cereal, vegetables, and fruits. 50 Mothers has a critical role in providing various diet and also in controlling their dietary intakes, preventing a high amount of non-nutritious diet. 8

This review shows a high sugar intake in children before the age of 6 months. 22 31 32 34 36 39 This review also found various sugary consumption with uncontrolled intakes. Solid foods are recommended after 6 months because motoric control has developed optimally to preserve feeding practice. Early sugary consumption can reduce the quantity and quality of breastfeeding and complementary feeding practices. Children tend to choose sugary foods over nutritious foods, which can disrupt healthy dietary patterns. 51 The high prevalence of stunting and ECC in this review can also be explained by the combination of limited nutritious foods and consumption of high-sugar foods.

The high prevalence of stunting and ECC indicates the bidirectional relationship between nutritional deficiency and poor oral health. 39 Oral cavity abnormalities can result from chronic nutritional deficiency in the pre-eruptive period of the teeth. Maternal breastfeeding and feeding practices play a significant role in the occurrence of this nutritional deficiency. The abnormalities can manifest as enamel hypoplasia, hyposalivation, and tooth eruption delay, which has been known to be susceptible in children with malnutrition. 16 43

Another finding regarding the experience of oral pain in these children was associated as an indicator of caries severity. 26 27 28 33 35 36 38 40 Mouth pain can also result from tooth eruption and periodontal and oral diseases, which are also potential risks in children with malnutrition. This condition tends to lower children's appetite, which can be another potential cause of inadequate nutrition intake. 43

Most of the studies observed that the mother has a positive perception of their children's oral health; however, knowledge of oral health was found to be poor. 26 27 34 40 Mothers faced the problem of implementing oral hygiene practices due to false projection of their knowledge and perception. Mothers knew the importance of maintaining oral hygiene to prevent caries, but they did not know the proper methods. All related studies observed inadequate frequency of toothbrushing twice a day. 21 24 33 We also found low dental appointment rates, which can be linked with severe caries. Poor oral hygiene and feeding practices were interlinked by manifesting into each other. 41 The combination of poor oral hygiene and poor feeding practices without adequate prevention will increase the risk of caries and nutritional deficiency. 51

We have conducted the first research explaining the correlation between maternal parenting styles and stunting and oral health in children. The latest studies only examine this context in malnutrition without linking it specifically with stunting. The data in this review were presented through descriptive analysis of each maternal parenting style variable. This review can be implemented in the clinical environment to educate mothers about the importance of adequate feeding and good oral hygiene practices. In addition, a new prevention concept from the bidirectional relationship between stunting and ECC can encourage implementation of optimal maternal parenting styles. There were some limitations to this review. First, no statistical analysis was used, so it cannot support the correlation found by the descriptive analysis. Second, the sample sizes included were not specifically in the stunting population, so there were some potential disturbance factors from another nutritional status. Third, we could not find articles with complete variable of mother's parenting style, which must be identified for comparison with determining the correlation between parenting and the incidence of ECC and malnutrition.

Additionally, there is a need to conduct a randomized controlled trial in the specific stunted population with complete identification of all maternal parenting style variables. Finally, future research should be extended to more countries.

## Conclusion

This review found that maternal parenting styles in nutrition and oral health have an effect on the occurrence of stunting and ECC in children. The high prevalence of stunting and ECC among the selected studies can be the combination of prolonged breastfeeding, poor complementary feeding practice, high sugary intakes, low oral health knowledge, low frequency of toothbrushing twice a day, and inadequate dental appointment.
